# Functional tissue-engineered microtissue formed by self-aggregation of cells for peripheral nerve regeneration

**DOI:** 10.1186/s13287-021-02676-0

**Published:** 2022-01-10

**Authors:** Jian Zhang, Chaochao Li, Fanqi Meng, Yanjun Guan, Tieyuan Zhang, Boyao Yang, Zhiqi Ren, Xiuzhi Liu, Dongdong Li, Jinjuan Zhao, Jie Zhao, Yu Wang, Jiang Peng

**Affiliations:** 1 Institute of Orthopedics, First Medical Center of the Chinese PLA General Hospital, Beijing Key Lab of Regenerative Medicine in Orthopedics, Key Lab of Musculoskeletal Trauma and War Injuries, PLA, No. 28 Fuxing Road, Beijing, 100853 People’s Republic of China; 2 Department of Spine Surgery, Peking University People’s Hospital, No.11 Xizhimen South Street, Xicheng District, Beijing, 100044 People’s Republic of China; 3 Beijing Tsinghua Changgeng Hospital Affiliated to Tsinghua University, Tsinghua University Clinical School, Changping District, Beijing, 102218 People’s Republic of China; 4 Co-innovation Center of Neuroregeneration, Nantong University, Nantong, 226007 Jiangsu Province People’s Republic of China

**Keywords:** Microtissue, Adipose-derived mesenchymal stem cells, 3D-culture, Schwann cells, Dorsal root ganglion, Co-culture, Peripheral nerve regeneration, Tissue engineering

## Abstract

**Background:**

Peripheral nerve injury (PNI) is one of the essential causes of physical disability with a high incidence rate. The traditional tissue engineering strategy, Top-Down strategy, has some limitations. A new tissue-engineered strategy, Bottom-Up strategy (tissue-engineered microtissue strategy), has emerged and made significant research progress in recent years. However, to the best of our knowledge, microtissues are rarely used in neural tissue engineering; thus, we intended to use microtissues to repair PNI.

**Methods:**

We used a low-adhesion cell culture plate to construct adipose-derived mesenchymal stem cells (ASCs) into microtissues in vitro, explored the physicochemical properties and microtissues components, compared the expression of cytokines related to nerve regeneration between microtissues and the same amount of two-dimension (2D)-cultured cells, co-cultured directly microtissues with dorsal root ganglion (DRG) or Schwann cells (SCs) to observe the interaction between them using immunocytochemistry, quantitative reverse transcription polymerase chain reaction (qRT-PCR), enzyme-linked immunosorbent assay (ELISA). We used grafts constructed by microtissues and polycaprolactone (PCL) nerve conduit to repair sciatic nerve defects in rats.

**Results:**

The present study results indicated that compared with the same number of 2D-cultured cells, microtissue could secrete more nerve regeneration related cytokines to promote SCs proliferation and axons growth. Moreover, in the direct co-culture system of microtissue and DRG or SCs, axons of DRG grown in the direction of microtissue, and there seems to be a cytoplasmic exchange between SCs and ASCs around microtissue. Furthermore, microtissues could repair sciatic nerve defects in rat models more effectively than traditional 2D-cultured ASCs.

**Conclusion:**

Tissue-engineered microtissue is an effective strategy for stem cell culture and therapy in nerve tissue engineering.

**Supplementary Information:**

The online version contains supplementary material available at 10.1186/s13287-021-02676-0.

## Background

Peripheral nerve injury (PNI) is one of the important causes of physical disability with a high incidence rate. Epidemiological statistics show that patients with PNI account for 2.8% of trauma patients, and at least 2 million people worldwide suffer from PNI each year [1–3]. When there is a long segmental nerve defect, the effect of direct end-to-end anastomosis is poor due to high tension [4]. At present, autologous nerve transplantation is still the standard gold method for treating long segmental nerve defects, but it has some limitations, such as limited donor sources, complications from second operations, and permanent denervation dysfunction of donor site [5]. Therefore, effective autologous nerve replacement is critical in the field of peripheral nerve regeneration.

In recent years, the rapid development of tissue engineering technology provides a new therapeutic method for repairing PNI. Researchers used tissue-engineered nerve graft to replace autologous nerve to repair PNI and achieved promising therapeutic effect [6–9]. At present, tissue engineering technology has two main strategies: Top-Down strategy and Bottom-Up strategy. Traditional tissue engineering adopts the Top-Down strategy: the graft skeleton matching the size and shape of the recipient region is constructed through three-dimension (3D) printing and other methods, and then high-density seed cells with or without bioactive molecules are added to scaffold to construct the tissue engineered graft [10–12]. However, this strategy has some limitations. For example, traditional two-dimension (2D)-cultured cells require trypsin digestion before use, which can destroy the extracellular matrix (ECM) and the microenvironment for cell growth and reduce cell activity [13, 14]; cells were unevenly distributed in the scaffold, and cells in the central region were necrotic due to hypoxia and difficult excrement of metabolic waste [15, 16]; after transplantation to the recipient region, single cells are vulnerable to the interference of various factors, such as ischemia and inflammation, leading to cell loss or death [16].

To solve the problem of traditional tissue engineering strategies, a new tissue-engineered strategy, Bottom-Up strategy (tissue-engineered microtissue strategy), has recently emerged, that is, seed cells were firstly aggregated into three-dimensional microtissue by means of hanging-drop method, microwell plate, microfluidic, etc., and then these microtissues were assembled into a macroscopic tissue for damaged tissue repair, or directly applied to the target tissues. At present, there is no clear definition of microtissue, which usually refers to the microtissues aggregated by seed cells under the action of cell–cell or cell-ECM under the action of upside-down droplets, low-adhesion cell culture plates, microcarriers, microgels and other culture media [17]. Microtissues have several advantages: Compared with single cells, microtissues, as microtissues formed by the aggregation of cells, can protect cells from inflammation, ischemia and other factors after implantation in the body [18]; the structural characteristics of microtissues are similar to those of natural tissues, providing a good microenvironment for cell proliferation and differentiation [19–21]. During cell culture, cells in microtissues are always in 3D space, and this 3D culture method is conducive to improving cell activity [22, 23]. Microtissues can be directly injected into target tissues [19, 24, 25], or can replace traditional 2D-cultured cells combined with 3D scaffolds [26] or be mixed into bio-ink for 3D printing [27, 28], all of them have achieved promising repair effects. The microtissue strategy has made breakthroughs in bone tissue engineering [26, 29–33], cartilage tissue engineering [19, 24, 34–36], adipose tissue engineering [22, 28], myocardial tissue engineering [37–40], vascular tissue engineering [41, 42], oncology [43, 44], etc. However, to the best of our knowledge, microtissues are rarely used in neural tissue engineering; thus, we intended to use microtissues to repair PNI.

Seed cells are the core element of tissue engineering, determining the therapeutic effect of tissue-engineered grafts. Mesenchymal stem cells (MSCs) can secret various neurotrophic factors to promote nerve regeneration. Among them, adipose-derived mesenchymal stem cells (ASCs), like other stem cells, have good biological characteristics, are widely derived and are easy to culture. They are satisfactory seed cells for neural tissue engineering [22, 27, 45].

In this study, we constructed ASCs microtissues using low-adhesion cell culture plates and analyzed the physicochemical properties of the microtissues. To explore the effects of 2D and 3D culture methods on the activity and function of ASCs, we co-culture microtissues with dorsal root ganglion (DRG) or Schwann cells (SCs), respectively, to observe the proliferation of SCs and the axon growth of DRG. Next, we conducted in vivo experiments to test the repair effect of microtissue on rat sciatic nerve defect model. We hypothesized that the 3D culture method is conducive to improve cell activity, microtissue can promote SCs proliferation and DRG axon growth and achieve promising repair effect in the sciatic nerve defect model.

## Methods

### Animals

The animal experiments were approved by the Institutional Animal Care and Use Committee of the Chinese PLA General Hospital. Sprague–Dawley (SD) rats, including 12-h-old rats, 3-day-old rats, 3-day-old GFP-infected rats, and 12-week-old rats, were obtained from the Laboratory Animal Research Center of Chinese PLA General Hospital. After the transplantation, the rats were kept in a temperature-controlled room under a 14 h/10 h light/dark cycle.

### Cell isolation and culture

ASCs or GFP-ASCs were isolated from the groin adipose tissue of 3-day-old SD rats or GFP-infected SD rats following previously published protocols [46]. Briefly, adipose tissue was minced to 1 mm^3^ pieces and digested with collagenase type II (1.5 mg/ml; C6885; Sigma) dissolved in Dulbecco’s modified Eagle’s medium/Ham’s F-12 (DMEM/F-12; Corning) at 37 °C for 30 min. The cell suspension was filtered, centrifuged and plated in α-modified minimal essential medium (α-MEM) (SH30265; Hyclone) supplemented with 10% fetal bovine serum (FBS) (35-011-CV; Corning), and penicillin–streptomycin solution (10,000 U/ml; 15140122; Gibco). ASCs were maintained in a humidified 5% CO^2^-containing atmosphere at 37 °C. ASCs were used for subsequent experiments at passage 2 or 3.

SCs were isolated from the sciatic nerves of 3-day-old GFP-infected SD rats following previously published protocols [47]. Briefly, sciatic nerves were minced to 1 mm^3^ pieces and digested with collagenase NB4 (2 mg/ml; 17454; SERVA) dissolved in DMEM/F-12 at 37 °C for 15 min. Then, the cell suspension was filtered, centrifuged and re-suspended in DMEM/F-12 containing 10% FBS. GFP-SCs were maintained in a humidified 5% CO^2^-containing atmosphere at 37 °C. When GFP-SCs reached 80% confluency, they were purified by digesting with the collagenase NB4, through which fibroblasts are not digested. GFP-SCs were used for subsequent experiments at passage 2 or 3.

### Flow cytometry

P3 ASCs were resuspended following digestion with 0.25% trypsin and washed three times with phosphate-buffered saline (PBS). A minimum of 1 × 10^5^ cells were collected from each sample. Samples were incubated with Anti-CD34 antibody (NBP2-29455; Novus), Anti-CD45RA antibody (561886; BD Pharmingen), Anti-CD73 antibody (orb629433; BIORBYT), Anti-CD90 antibody (561973; BD Pharmingen), and Anti-CD105 antibody (NB500-452; Novus) in the dark at room temperature for 30 min. The samples were subjected to flow cytometry, and data were analyzed using Paint-A-Gate Pro™ software (FACSCalibur™; BD Biosciences).

### Fabrication and characterization of microtissues

#### Fabrication of microtissues

We used an ultra-low attachment microplate (4516; Corning) to construct microtissues. The microplates have 384 wells, the maximum volume of each well is 90 μl, and the unique hydrogel surface of each well inhibits cell attachment. Briefly, 70-μl cell suspension containing 1 × 10^4^ (or 0.75 × 10^4^ or 0.5 × 10^4^) cells were transferred into each well, and place the cell culture plates in a humidified 5% CO_2_-containing atmosphere at 37 °C. Microtissues containing 1 × 10^4^ cells were collected 72 h later for subsequent experiments.

#### Morphological observations

In this low-adhesion microplate, only one microtissue can be produced per well. We constructed three types of microtissues with different numbers of cells (1 × 10^4^, 0.75 × 10^4^, and 0.5 × 10^4^ cells). The morphology and diameter of microtissues were regularly observed for 7 days and photographed.

#### Immunofluorescence staining

Immunofluorescence staining was performed to determine the composition of microtissues. The microtissues were fixed with 4% paraformaldehyde, and then dehydrated with 20% and 30% sucrose, respectively. Before staining, the microtissues were cut into 9-μm flakes using a frozen slicer. Subsequently, the samples were blocked with 10% goat serum (SL038; Solarbio) for 1 h at room temperature after washing with PBS. Then, the samples were incubated overnight at 4 °C with Mouse anti-laminin antibody (1:200; ab 242198; Abcam) and Rabbit anti-fibronectin antibody (1:500; ab268020; Abcam). Next, they were incubated with Goat Anti-Mouse IgG H&L (Alexa Fluor® 488; 1:200; ab150117; Abcam) and Goat Anti-Rabbit IgG H&L (Alexa Fluor® 594; 1:200; ab150084; Abcam) secondary antibodies for 2 h at room temperature. 4′,6-diamidino-2-phenylindole dihydrochloride (DAPI) was used for 10 min at room temperature to stain the nuclei. Images were taken using a fluorescence microscope (PANNORAMIC Confocal; 3DHISTECH).

### Cell viability evaluation

A live/dead viability assay was performed to evaluate cell viability. Microtissues were harvested, washed with PBS and stained using a cell viability assay kit (L7010; Invitrogen), and cell viability of microtissues was evaluated using a fluorescence microscope (PANNORAMIC Confocal; 3DHISTECH).

### Effects of 2D and 3D culture on cell characteristics

#### Morphological observations

For 2D-cultured ASCs and 3D-cultured ASCs (microtissues), 0.25% trypsin was used to collect cells. Briefly, for 2D cell suspension, we need to pour out the waste medium in the cell culture bottle, add trypsin and shake for 10 s till full digestion, add fresh medium to stop digestion and collect 2D cell suspension. For 3D cell suspension, the experimental procedure is the same, except that it needs to be shaken with an oscillator in a 37 °C incubator for 6 min to fully digest. The morphology of 2D- and 3D-cultured cells was observed and photographed using an optical microscope. The cell diameter was measured using Image-Pro Plus software.

#### Real‐time quantitative reverse transcription PCR (Real‐time qRT‐PCR)

Total RNA from 2D-cultured cells and 3D microtissues was extracted with TRIzol® reagent (15596018; Invitrogen) for gene expression analysis. cDNA was synthesized using ReverTra Ace® qPCR RT Kit (FSQ-101; TOYOBO). Quantitative PCR was performed with a StepOne™ Real-Time PCR System (Applied Biosystems). The relative gene expression was normalized to the glyceraldehyde 3-phosphate dehydrogenase (GAPDH) expression and is presented as the fold-change using the 2^−ΔΔCT^ method. The relative expression values were further normalized to the respective sample of 2D-cultured cells. All experiments were repeated three times independently. Table 1 lists the primers designed for qPCR.

**Table 1:** List of primer sequences used in this study

Target genes	Forward primer (5′–3′)	Reverse primer (5′–3′)	Annealing temperature (°C)
GAPDH	ATGGTGAAGGTCGGTGTGAACG	TTACTCCTTGGAGGCCATGTAG	55–62
BDNF	TGGAACTCGCAATGCCGAACTAC	TCCTTATGAACCGCCAGCCAATTC	57
VEGF	GGCTCACTTCCAGAAACACG	GTGCTCTTGCAGAATCTAGTGG	55
IL-4	CAAGGAACACCACGGAGAACGAG	CTTCAAGCACGGAGGTACATCACG	56
IL-10	CTGCTCTTACTGGCTGGAGTGAAG	TGGGTCTGGCTGACTGGGAAG	56
IL-13	CTCGCTTGCCTTGGTGGTCTTG	GCACAGGGAAGTCTTCTGGTCTTG	56

*GAPDH* glyceraldehyde 3-phosphate dehydrogenase, *BDNF* brain-derived neurotrophic factor, *VEGF* vascular endothelial growth factor, *IL-4* interleukin-4, *IL-10* interleukin-10, *IL-13* interleukin-13

#### Enzyme-linked immunosorbent assay (ELISA)

To measure the concentration of brain-derived neurotrophic factor (BDNF), nerve growth factor (NGF), vascular endothelial growth factor (VEGF), interleukin 13 (IL-13) of 2D-cultured cells and 3D microtissues, the culture supernatants of the two groups with the same number of cells (4 × 10^5^ cells) were collected 72 h later and centrifuged at 1500 rpm at 4 °C for 10 min. Then, the supernatants were analyzed by ELISA using Rat BDNF ELISA Kit (MEIMIAN; MM-0209R1), Rat NGF ELISA Kit (MEIMIAN; MM-0187R1), Rat VEGF ELISA Kit (MEIMIAN; MM-0807R1), Rat IL-13 ELISA Kit (MEIMIAN; MM-0085R1), respectively. The absorbances of each well at 450 nm were determined using a Microplate Reader (Labsystems Multiskan). All experiments were repeated three times independently.

### Microtissues were co-cultured with Schwann cells (SCs) or dorsal root ganglion (DRG)

#### Microtissues were co-cultured with SCs directly and indirectly

For exploring the effect of paracrine of microtissues on SCs, GFP-SCs were co-cultured indirectly with microtissues (Microtissue group) or 2D-cultured ASCs (2D group) or cell-free medium (Control group) in a Transwell® system (3450; Corning) consisting of six inserts, each containing a polyester membrane with a diameter of 24 mm and an aperture of 0.4 μm (Additional file 1: Figure S1). Specifically, each lower chamber was seeded with 2 × 10^4^ GFP-SCs, then 50 microtissues (each microtissue contains 1 × 10^4^ cells) or 5 × 10^5^ 2D-cultured ASCs or cell-free medium were seeded in the upper inserts, respectively. Moreover, adequate DMEM/F-12 containing 2% FBS to each culture system. After co-culture for 3 days, GFP-SCs were observed using a fluorescence microscope (PANNORAMIC Confocal; 3DHISTECH) and photographed. At 10× magnification, ten fields were randomly selected from each group, and cell counts were performed using Image-Pro Plus software.

Before evaluating the interaction between microtissues and SCs, ASCs was labeled with fluorescent dye PKH26 (MINI26; Sigma), according to the manufacturer’s protocol. Briefly, after digestion and centrifugation, ASCs were resuspended with dilution buffer, and the labeling solution consists of 4-μl fluorescent dye, and 1-ml dilution buffer was added. After 4 min, 2-ml FBS was added to terminate staining, and the cells were cleaned with medium 3 times. After pKH26-ASCs was constructed into microtissues, we co-cultured the microtissues with GFP-SCs directly. Specifically, we mixed 50 microtissues with 2 × 10^4^ GFP-SCs and placed them in a cell culture dish with adequate DMEM/F-12 containing 2% FBS. After 3 days of co-culture, a fluorescence microscope (PANNORAMIC Confocal; 3DHISTECH) was used to observe and photograph.

#### Microtissues were co-cultured with DRG directly and indirectly

Prior to co-culture, we extracted the DRG following previously published protocols [47]. Briefly, the spine of 12-h-old SD rats was completely removed and dissected into two halves along the mid-sagittal plane. Using a microscope, the DRG was removed from the ambilateral intervertebral foramen and the outer membrane was carefully removed. DRG were co-cultured indirectly with microtissues (Microtissue group) or 2D-cultured ASCs (2D group) or cell-free medium (Control group) in a transwell system. Specifically, the lower chamber was seeded with DRG, then 50 microtissues (each microtissue contains 1 × 10^4^ cells) or 50 × 10^4^ 2D-cultured ASCs or cell-free medium were seeded in the upper inserts, respectively. Neurobasal®-A Medium (10888–022; Gibco) supplemented with GlutaMAX™ Supplement (1:100; 35,050-061; Gibco) and B-27® Serum-Free Supplement (1:50; 17,504-044; Gibco) was used to co-culture the three groups. After co-culture for 7 days, immunofluorescence staining of S100 (primary antibody: Rabbit anti-S100 antibody (1: 200; S2644; Sigma), secondary antibody: Goat Anti-Rabbit IgG H&L (Alexa Fluor® 594; 1:200; ab150084; Abcam) and neurofilament protein 200 (NF200) (primary antibody: Mouse anti-Neurofilament 200 antibody (1:800; N0142; Sigma), secondary antibody: Goat Anti-Mouse IgG H&L (Alexa Fluor® 488; 1:200; ab150117; Abcam)) was performed to stained the SCs and axon of the DRG, and DAPI was used to stained the nuclei. Images were taken using a fluorescence microscope (PANNORAMIC Confocal; 3DHISTECH). Four DRG were randomly selected from each group for statistical analysis. The two longest axons per quadrant (a total of four quadrants) were measured and used to calculate the mean axons length of each DRG using Image-Pro Plus software.

To explore the interaction between microtissues and DRG, we co-cultured the GFP-ASCs microtissues with DRG directly. Specifically, we mixed 50 GFP-ASCs microtissues with 5 DRG and placed them in a cell culture dish with adequate Neurobasal®-A Medium containing GlutaMAX™ Supplement and B-27® Serum-Free Supplement. After 7 days of co-culture, immunofluorescence staining of NF200 (primary antibody: Mouse anti-Neurofilament 200 antibody (1:800; N0142; Sigma), secondary antibody: Goat Anti-Mouse IgG H&L (Alexa Fluor® 594; 1:200; ab150116; Abcam)) was performed to evaluate the interaction between microtissues and DRG, and DAPI was used to stained the nuclei. Images were taken using a fluorescence microscope (PANNORAMIC Confocal; 3DHISTECH).

### In vivo peripheral nerve defect repair

#### Microtissue transplantation for the treatment of peripheral nerve defect

Forty female SD rats, 12 weeks old and weighing 250–300 g, were used for in vivo experiments. Rats were anesthetized with 3% sodium pentobarbital solution (30 mg/kg body weight), and 10-mm sciatic nerve defects were created using a microsurgery scissor. The rats were randomly divided into four groups (*n* = 10 per group) according to the different types of nerve graft used to bridge the sciatic nerve defect: (a) Hollow group, in which the sciatic nerve defect was bridged with polycaprolactone (PCL) nerve conduit, and the cell-free medium was injected into the conduit; (b) 2D group, in which the 50 × 10^4^ ASCs was injected into the PCL nerve conduit; (c) Microtissue group, in which the 50 microtissues was injected into the PCL nerve conduit; (d) ANG group, in which an autologous nerve graft was applied. Fibrin gel was used to seal the suture interface. For a detailed process of PCL nerve conduit, please refer to our previous study [48].

#### Gait analysis

Gait analysis was performed at 4 weeks, 8 weeks and 12 weeks after transplantation to evaluate the recovery of sciatic nerve function. Briefly, footprints were collected from each group using a CatWalk footprint analysis system (XT 10.6; Noldus). At least three approved runs were required for each rat in each group for each time point in the analysis. Sciatic nerve function index (SFI) was obtained using Noldus Catwalk Analysis software.

#### Electrophysiology

At the 12th week post-transplantation, a fully functional electromyography machine performed neural electrophysiological evaluation on six rats in each group (Keypoint; Medtronic). Briefly, exposing the nerve graft along the original incision, the stimulating electrode was placed on the proximal end of the graft and the recording electrode was placed inside the abdomen of the gastrocnemius muscle, and then the compound muscle action potentials (CMAPs) were induced and recorded. Meanwhile, CMAPs of the contralateral normal sciatic nerve also need to be recorded. The parameters of electrical stimulation were 3.0 mA and 1 Hz. The recovery of nerve conduction function was evaluated by the peak amplitude and latency of CMAPs.

### Histological evaluation of nerve grafts

To observe the nerve growth in the early stage, four rats in each group were sacrificed by intraperitoneal injection of a sodium pentobarbital overdose at 4th week post-transplantation. The nerve grafts were carefully removed and fixed with 4% paraformaldehyde, and then dehydrated with 20% and 30% sucrose, respectively. Before staining, the nerve grafts were sliced longitudinally into 9-μm slices using a frozen slicer. Then, the samples were incubated overnight at 4 °C with primary antibodies against S100 (Rabbit anti-S100 antibody (1: 200; S2644; Sigma)) and NF200 (Mouse anti-Neurofilament 200 antibody (1:800; N0142; Sigma)). Next, they were incubated with secondary antibodies (Goat Anti-Rabbit IgG H&L (Alexa Fluor® 594; 1:200; ab150084; Abcam) and Goat Anti-Mouse IgG H&L (Alexa Fluor® 488; 1:200; ab150117; Abcam)) for 2 h at room temperature. DAPI was used to stained for 10 min at room temperature to label the nuclei. Images were taken using a fluorescence microscope (PANNORAMIC Confocal; 3DHISTECH).

At the 12th week post-transplantation, the nerve grafts of all rats were carefully removed. The distal part of the nerve graft was dissected for staining of toluidine blue and observation of transmission electron microscopy (TEM) to evaluate the amount and thickness of myelin sheath. Briefly, after fixation, the distal section of nerve graft was sectioned into transverse semi-thin sections of 1-μm thickness using an ultramicrotome (EM UC7; Leica), and then the samples were stained with Toluidine Blue O solution (1% in sodium borate; G3663; Solarbio). Six 100× magnification visual fields were randomly selected from each group to count the mean density of myelinated nerve fibers by Image-Pro Plus software. Subsequently, the distal section of nerve graft was cut into transverse ultrathin sections of 70-nm thickness using an ultramicrotome (EM UC7; Leica). Then, the samples were counterstained with 3% lead citrate and uranyl acetate, and observed using TEM (CM-120; Philips). Ten 2000× magnification visual fields were randomly selected from each group to measure the myelin sheath thickness by Image-Pro Plus software. The middle segment of the nerve graft was transversally cut into 9-μm slices, and the remaining intact nerve grafts were sliced longitudinally into 9-μm slices using a frozen slicer, and then performed the immunofluorescence staining. Briefly, samples were incubated with primary antibodies against S100 and NF200 (Rabbit anti-S100 antibody (1: 200; S2644; Sigma)) and Mouse anti-Neurofilament 200 antibody (1:800; N0142; Sigma), secondary antibodies (Goat Anti-Rabbit IgG H&L (Alexa Fluor® 488; 1:200; ab150077; Abcam) and Goat Anti-Mouse IgG H&L (Alexa Fluor® 594; 1:200; ab150116; Abcam)) and DAPI, successively. Images were taken using a fluorescence microscope (PANNORAMIC Confocal; 3DHISTECH).

### Histological evaluation of gastrocnemius

At 12-week post-transplantation, bilateral gastrocnemius muscles of rats were removed after electrophysiological evaluation and weighed immediately to determine the muscle weight ratio (injured side/normal side). The belly of gastrocnemius muscle was fixed in 4% paraformaldehyde for two hours, and then transverse paraffin sections with a thickness of 10 μm were performed and stained with modified Masson’s trichrome stain kit (G1345; Solarbio). At 20× magnification, six fields were randomly selected from each group, and the mean cross-sectional area of gastrocnemius fibers was measured using Image-Pro Plus software.

### Statistical analysis

All data are expressed as the means ± standard deviation (SD). Statistical analysis was performed using GraphPad Prism 8. Student’s t test was used to compare differences between two groups, and one-way analysis of variance (ANOVA) was used to compare differences between multiple groups. Differences between groups were considered significant at *p < 0.05, **p < 0.01, ***p < 0.001 and ****p < 0.0001.

## Results

### Characterization of ASCs

Flow cytometry showed that the stem cell markers CD73 (87.35%), CD90 (99.96%), and CD105 (95.14%) were generally highly expressed in ASCs, and less than 2% of ASCs were immunopositive for the hematopoietic stem cell markers CD34, CD45 (Additional file 2: Figure S2).

### Characterization of microtissues

**Fig. 1:**
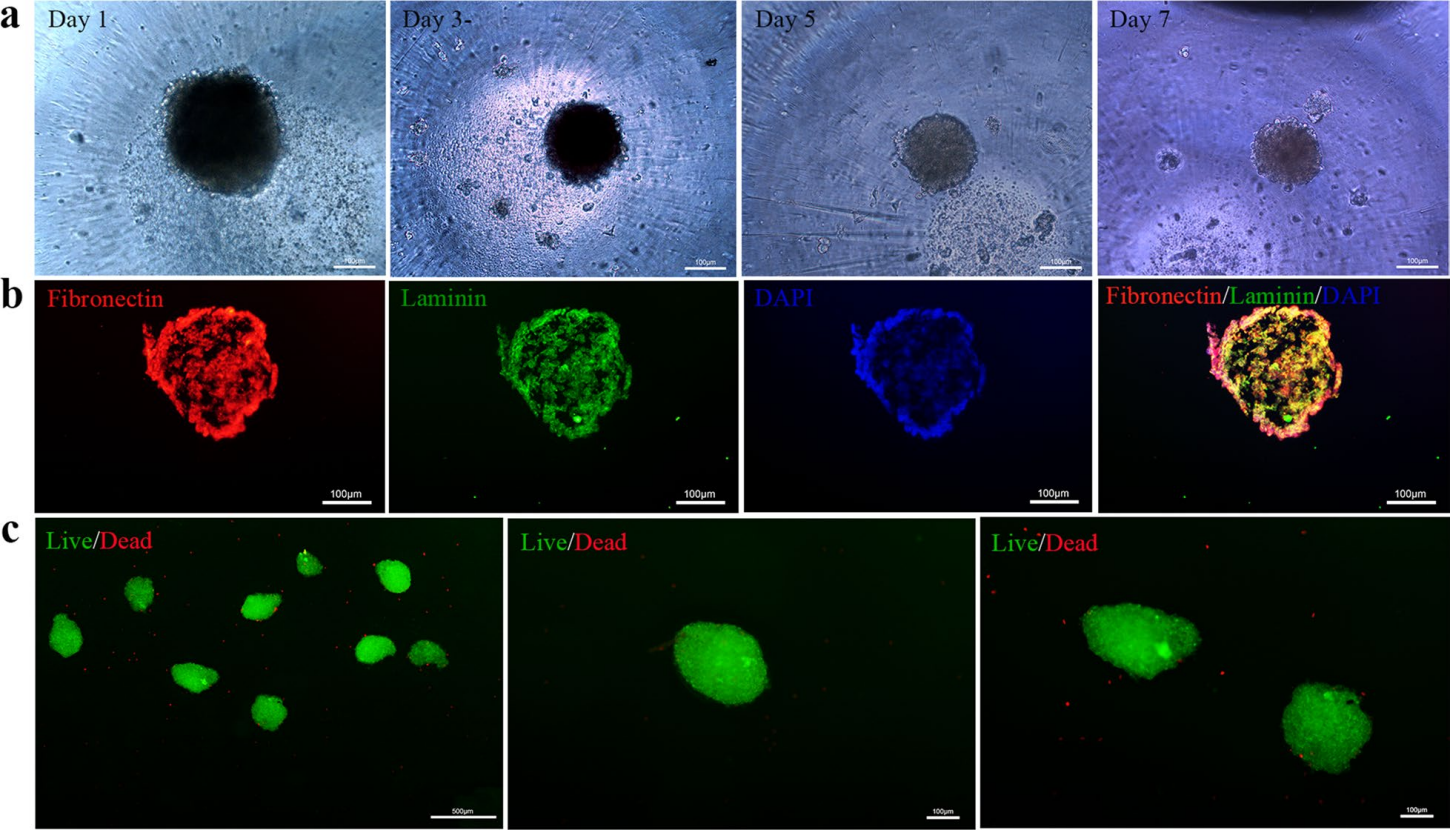
Characterization of microtissues. **a** The morphological changes of the microtissues were vigorously observed for 7 days. Scale bars: 100 μm. **b** The laminin exhibited green fluorescence, and the fibronectin exhibited red fluorescence, all nuclei showed blue fluorescence. Scale bars: 100 μm. **c** Live/dead staining showed that the majority of ASCs remained viable. Scale bars: 500 μm (left) and 100 μm (middle and right)

Three concentrations of cell suspension (1 × 10^4^ or 0.75 × 10^4^ or 0.5 × 10^4^ cells per 70 μl) were used to construct the microtissues, and the morphological changes of the microtissues (containing 1 × 10^4^ cells per microtissue) were vigorously observed for 7 days (Fig. 1a). The morphology of microtissues was smooth and spherical, and its diameter decreased with the extension of culture time. However, there was no statistical difference in the diameter of microtissues with different cell numbers (5000 cells/microtissue or 7500 cells/microtissue or 10,000 cells/microtissue). (Additional file 3: Figure S3). Immunofluorescence analysis revealed that the laminin exhibited green fluorescence, and the fibronectin exhibited red fluorescence, and all nuclei showed blue fluorescence (Fig. 1b). Cell viability was evaluated by live/dead staining, which showed that the majority of ASCs remained viable (Fig. 1c).

### Effects of 2D and 3D culture on cell characteristics

**Fig. 2:**
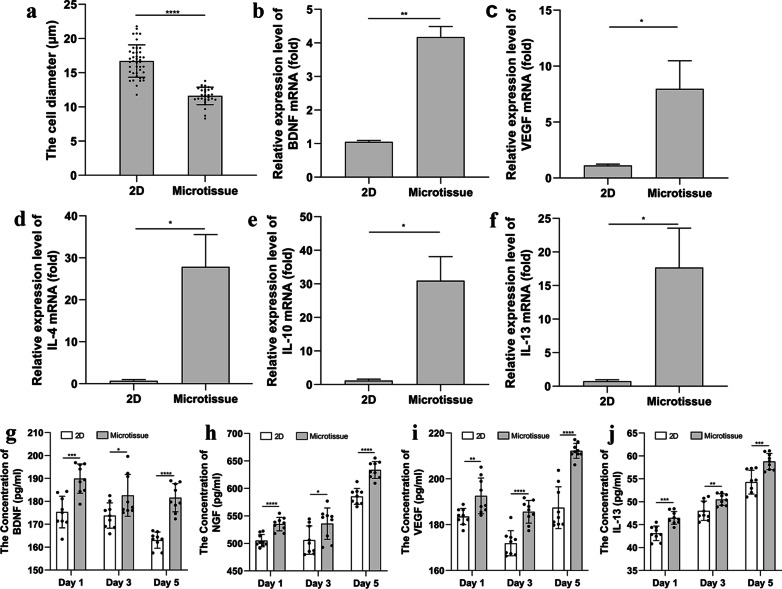
Effects of 2D and 3D culture on cell characteristics. **a** Compared with 2D-cultured ASCs, the diameter of 3D-cultured ASCs was relatively uniform and was reduced by 30.5% (*p* < 0.0001). **b** The relative mRNA expression levels of BDNF was significantly higher in the Microtissue group than 2D group (*p* < 0.01). **c** The relative mRNA expression levels of VEGF was significantly higher in the Microtissue group than 2D group (*p* < 0.05). **d** The relative mRNA expression levels of IL-4 was significantly higher in the Microtissue group than 2D group (*p* < 0.05). **e** The relative mRNA expression levels of IL-10 was significantly higher in the Microtissue group than 2D group (*p* < 0.05). **f** The relative mRNA expression levels of IL-13 was significantly higher in the Microtissue group than 2D group (*p* < 0.05). **g** The concentrations of BDNF secreted by 3D-cultured ASCs were significantly higher than 2D-cultured ASCs on day 1, day 3 and day 5, respectively (Day 1, *p* < 0.001; Day 3, *p* < 0.05; Day 5, *p* < 0.0001). **h** The concentrations of NGF secreted by 3D-cultured ASCs were significantly higher than 2D-cultured ASCs on day 1, day 3 and day 5, respectively (Day 1, *p* < 0.0001; Day 3, *p* < 0.05; Day 5, *p* < 0.0001). **i** The concentrations of VEGF secreted by 3D-cultured ASCs were significantly higher than 2D-cultured ASCs on day 1, day 3 and day 5, respectively (Day 1, *p* < 0.01; Day 3, *p* < 0.0001; Day 5, *p* < 0.0001). **j** The concentrations of IL-13 secreted by 3D-cultured ASCs were significantly higher than 2D-cultured ASCs on day 1, day 3 and day 5, respectively (Day 1, *p* < 0.001; Day 3, *p* < 0.01; Day 5, *p* < 0.001)

2D- and 3D-cultured ASCs were digested, cell morphology was observed and photographed using a light microscope, and cell diameter was measured with Image-Pro Plus software. The results showed that the cell diameter of 2D ASCs varied greatly (varies between 12 and 21 μm), and the average cell diameter was 16.70 ± 2.365 μm. The cell diameter of 3D ASCs was relatively uniform (varies between 8 and 13 μm), the mean cell diameter was 11.61 ± 1.289um, which was reduced by 30.5%, *p* < 0.0001 (Fig. 2a).

qRT-PCR was conducted to determine the relative mRNA expression levels in each group of cells. The relative mRNA expression levels of brain-derived neurotrophic factor (BDNF), a neurotrophic factor, were significantly higher in the Microtissue group than 2D group (*p* < 0.01) (Fig. 2b). The relative mRNA expression levels of vascular endothelial growth factor (VEGF), an angiogenic factor, was significantly higher in the Microtissue group than 2D group (*p* < 0.05) (Fig. 2c). The relative mRNA expression levels of anti-inflammatory factors, including IL-4, IL-10, and IL-13, were significantly upregulated in the Microtissue group compared with the 2D group (*p* < 0.05) (Fig. 2d–f).

The neurotrophins, angiogenic factors and inflammatory factors secreted by the two groups of cells were measured at the protein level by ELISA. The levels of BDNF secreted by 3D-cultured ASCs (Day1 189.90 ± 6.39 pg/ml; Day 3 182.60 ± 9.13 pg/ml; Day 5 181.60 ± 6.16 pg/ml) were significantly higher than 2D-cultured ASCs (Day1 175.30 ± 6.89 pg/ml; Day 3 173.80 ± 5.54 pg/ml; Day 5 163.00 ± 3.53 pg/ml) on day 1, day 3 and day 5, respectively (Day 1, *p* < 0.001; Day 3, *p* < 0.05; Day 5, *p* < 0.0001) (Fig. 2g). In addition, nerve growth factor (NGF) secretion levels also showed similar trends. The concentrations of NGF secreted by 3D-cultured ASCs (Day1 535.00 ± 12.28 pg/ml; Day 3 535.80 ± 28.54 pg/ml; Day 5 633.60 ± 15.35 pg/ml) were significantly higher than 2D-cultured ASCs (Day1 505.00 ± 11.16 pg/ml; Day 3 506.00 ± 25.79 pg/ml; Day 5 585.40 ± 14.53 pg/ml) on day 1, day 3 and day 5, respectively (Day 1, *p* < 0.0001; Day 3, *p* < 0.05; Day 5, *p* < 0.0001) (Fig. 2h). The levels of VEGF secreted by 3D-cultured ASCs (Day1 192.60 ± 7.82 pg/ml; Day 3 185.60 ± 4.92 pg/ml; Day 5 212.20 ± 3.26 pg/ml) were significantly higher than 2D-cultured ASCs (Day1 183.50 ± 3.52 pg/ml; Day 3 171.90 ± 5.54 pg/ml; Day 5 187.40 ± 9.12 pg/ml) on day 1, day 3 and day 5, respectively (Day 1, *p* < 0.01; Day 3, *p* < 0.0001; Day 5, *p* < 0.0001) (Fig. 2i). The levels of IL-13 secreted by 3D-cultured ASCs (Day1 46.47 ± 1.38 pg/ml; Day 3 50.49 ± 1.25 pg/ml; Day 5 58.79 ± 1.77 pg/ml) were significantly higher than 2D-cultured ASCs (Day1 43.11 ± 1.59 pg/ml; Day 3 48.02 ± 2.11 pg/ml; Day 5 54.28 ± 2.59 pg/ml) on day 1, day 3 and day 5, respectively (Day 1, *p* < 0.001; Day 3, *p* < 0.01; Day 5, *p* < 0.001) (Fig. 2j).

### Interactions between microtissue and SCs and DRG

**Fig. 3:**
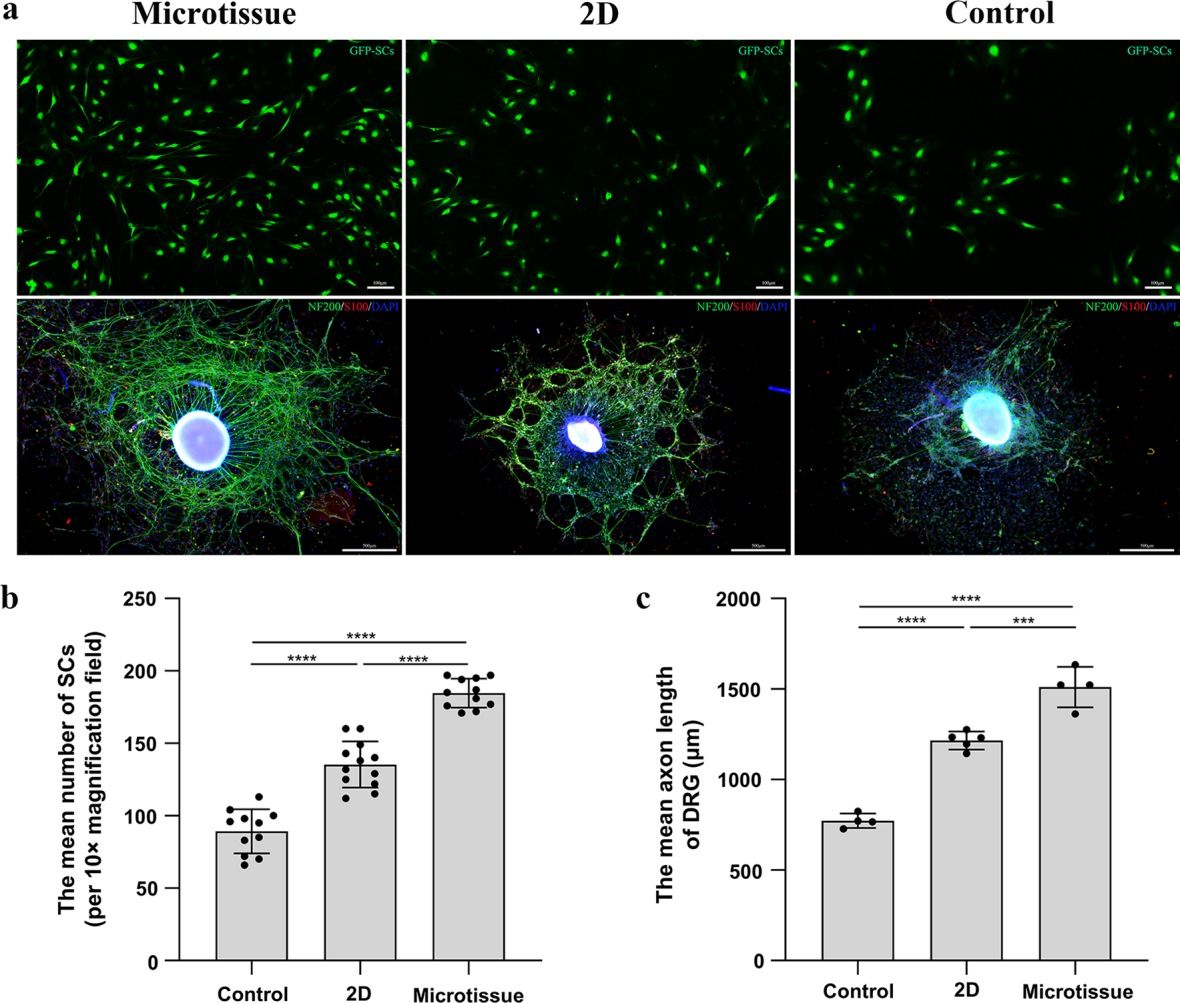
Indirectly co-culture each group and GFP-SCs or DRGs by a transwell system. **a** The up row is the indirect co-culture of each group with GFP-SCs. GFP-SCs exhibited green fluorescence. The bottom row is the indirect co-culture of each group with DRGs. The axons of DRG exhibited green fluorescence, SCs that migrated from the DRG exhibited red fluorescence, and all nuclei showed blue fluorescence. Scale bars: 100 μm (up row) and 500 μm (bottom row). **b** The number of SCs (per ×10 magnification field) in the Microtissue group was significantly higher than the 2D group (*p* < 0.0001) and the Control group (*p* < 0.0001). **c** The axon length of DRG in the Microtissue group was significantly longer than that in the 2D group (*p* < 0.001) and the Control group (*p* < 0.0001)

After indirectly co-culture microtissues and GFP-SCs using a transwell system for 3 days, SCs were photographed and counted (Fig. 3a). The number of SCs in the Microtissue group (184.70 ± 9.99 per 10× magnification field) was significantly higher than the 2D group (135.40 ± 15.91 per 10× magnification field) and the Control group (89.27 ± 15.23 per 10× magnification field) (microtissue VS 2D, *p* < 0.0001; microtissue VS control, *p* < 0.0001). The number of SCs in the 2D group was significantly higher than the Control group (*p* < 0.0001) (Fig. 3b). DRG were co-cultured with microtissues indirectly by a transwell system for 7 days, and immunofluorescence staining was performed on DRG. Immunofluorescence analysis showed that the axons of DRG exhibited green fluorescence, SCs that migrated from the DRG exhibited red fluorescence, and all nuclei showed blue fluorescence (Fig. 3a). The axon length of DRG in the Microtissue group (1510.00 ± 111.50 μm) was significantly longer than that in the 2D group (1216.00 ± 49.41 μm) and the Control group (771.90 ± 40.01 μm) (microtissue VS 2D, *p* < 0.001; microtissue VS control, *p* < 0.0001). The axon length of DRG in the 2D group was significantly longer than the Control group (*p* < 0.0001) (Fig. 3c).

**Fig. 4:**
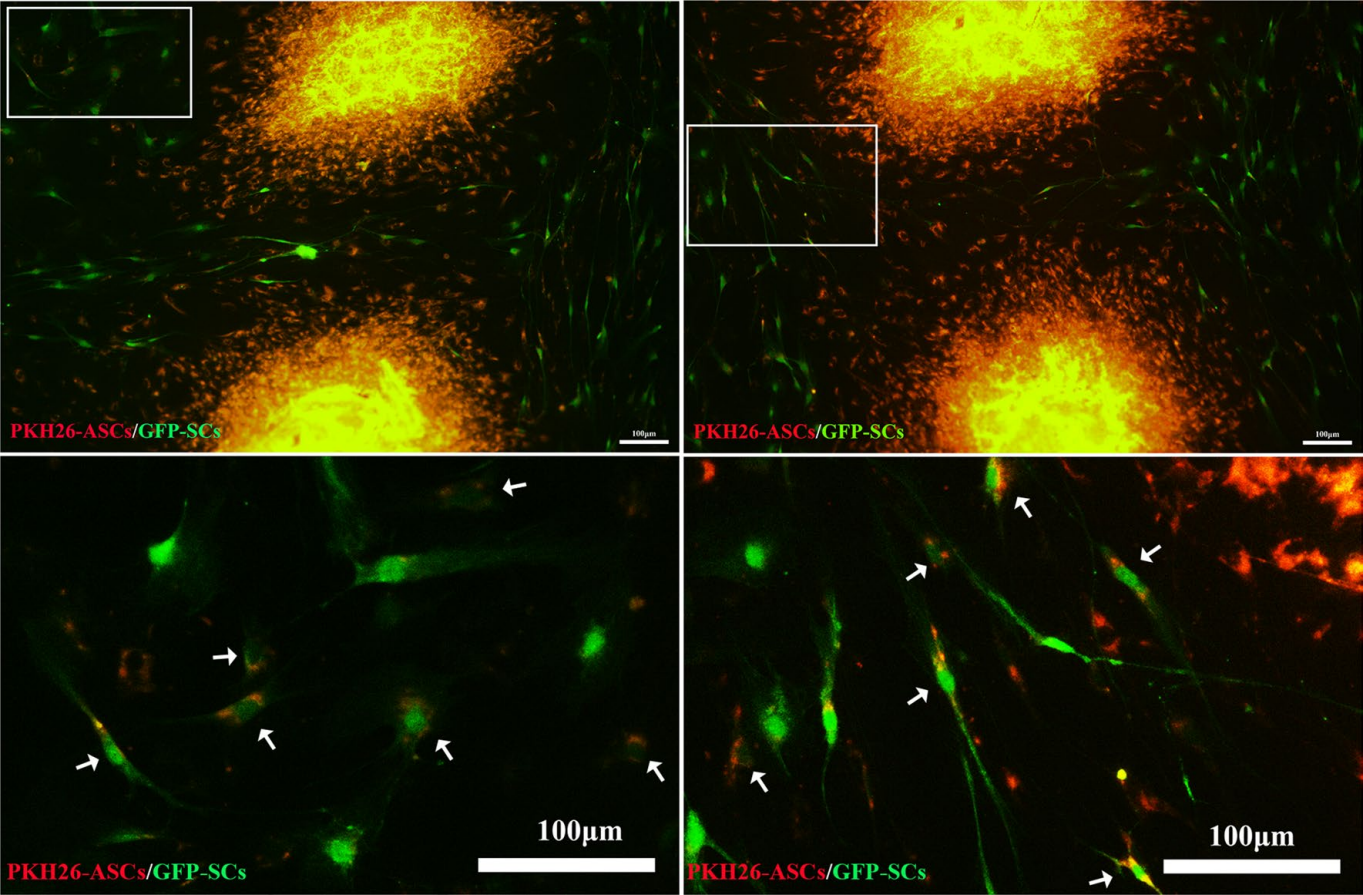
Directly co-culture microtissues and GFP-SCs. GFP-SCs exhibited green fluorescence and PKH26-ASCs microtissues exhibited red fluorescence. Photographs in the bottom row are higher magnification views of those in the box in the up row. However, some GFP-SCs around the microtissues showed red fluorescence, there seems to be cytoplasmic exchange between microtissues and SCs (white arrow). Scale bars: 100 μm

**Fig. 5:**
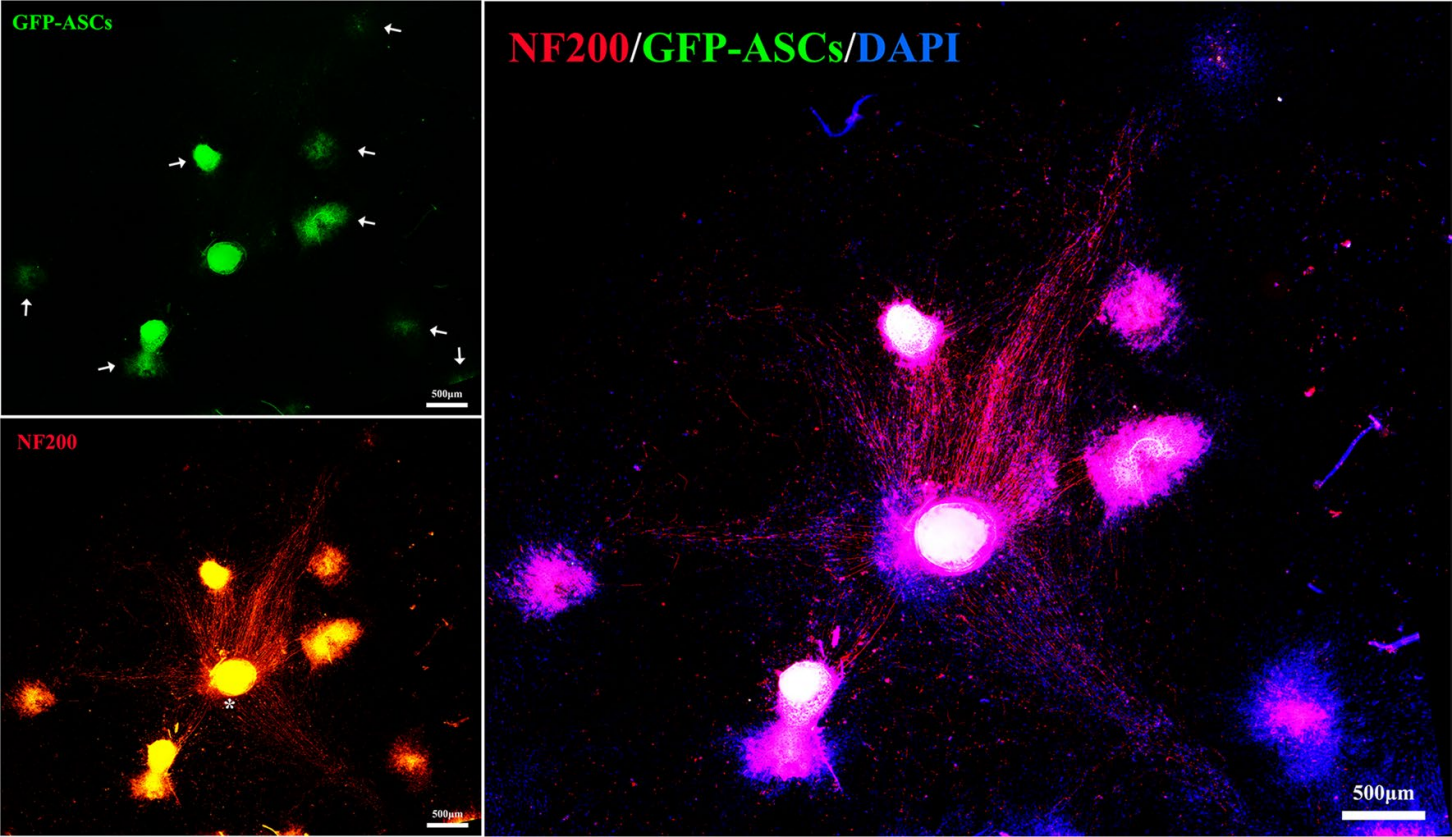
Directly co-culture GFP-ASCs microtissues and DRG. GFP-ASCs microtissues exhibited green fluorescence, the axons of DRG exhibited red fluorescence, and all nuclei showed blue fluorescence (white arrow: GFP-ASCs microtissues; White asterisk: DRG). The axons of the DRG grow in the microtissues direction, and the axon length is much longer than that of DRG in all the above experiments. Scale bars: 500 μm

After directly co-culture PKH26-ASCs microtissues and GFP-SCs for 3 days, immunofluorescence analysis showed that GFP-SCs exhibited green fluorescence, and PKH26-ASCs microtissues exhibited red fluorescence. However, some GFP-SCs around the microtissues showed red fluorescence, there seems to be cytoplasmic exchange between microtissues and SCs (Fig. 4). DRG were co-cultured with GFP-ASCs microtissues directly for 7 days, immunofluorescence staining was performed on DRG. Immunofluorescence analysis showed that GFP-ASCs microtissues exhibited green fluorescence, the axons of DRG exhibited red fluorescence, and all nuclei showed blue fluorescence. The axons of the DRG grow in the microtissues direction, and the axon length is much longer than that of DRG in all the above experiments (Fig. 5). These results indicate that direct interaction between DRG and microtissue can promote the growth of DRG axons.

### Functional recovery of the sciatic nerve following microtissue transplantation

**Fig. 6:**
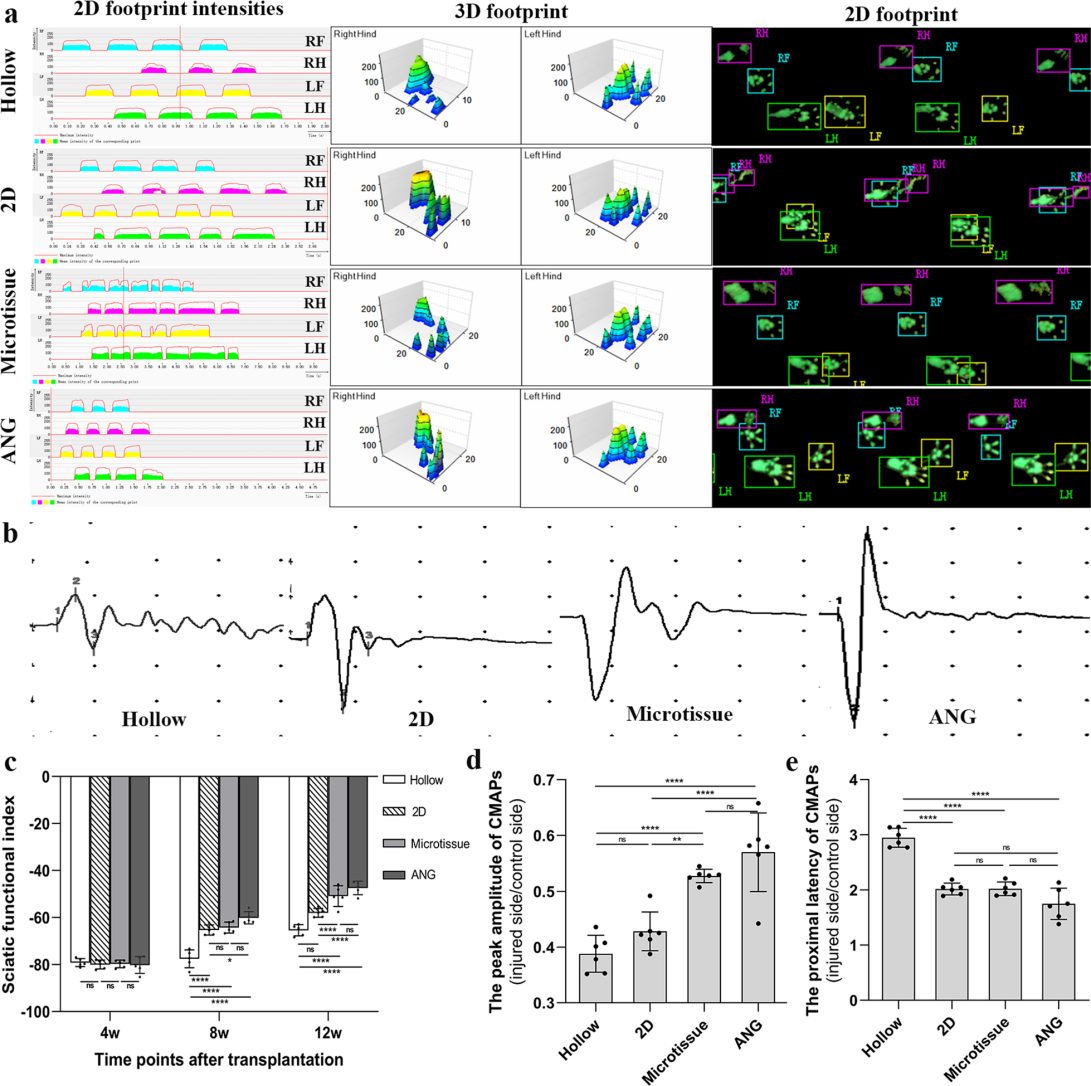
Gait analysis and electrophysiological examination after transplantation. **a** Representative 2D footprint (right), 3D footprint (middle), and 2D footprint intensity (left) of each group at the 12th week after transplantation. **b** Representative CMAPs oscillograms of each group at the 12th week after transplantation. **c** The SFI of 2D, Microtissue, and ANG group were significantly higher than Hollow group at the 8th week after transplantation. At the 12th week after transplantation, the SFI of the Microtissue group was significantly better (*p* < 0.0001) than 2D group. **d** The peak amplitude ratio of CMAPs in the Microtissue group was significantly higher (*p* < 0.01) than the 2D group. **e** The latency ratio of CMAPs in the Hollow group was significantly longer (*p* < 0.0001) than the 2D group, Microtissue group, and ANG group, and there was no statistical difference between the latter three groups

Gait analysis was used to evaluate the recovery of sciatic nerve function. At the 12th week after transplantation, representative footprint view, 2D and 3D stress diagrams of each group are shown in Fig. 6a. As seen from the 2D stress diagrams, the period of touching the ground of the right hind (RH) leg (injured side) was markedly shorter than that of the left hand (LH) leg (normal side), and the pressure intensity on the ground of RH leg was significantly less than that of LH leg in Hollow and 2D group. However, there is no significant difference in period and pressure intensity of touching the ground between RH leg and LH leg in microtissue and ANG group (Fig. 6a). According to the footprint views and 3D stress diagrams, the extension of the RH toes in Microtissue group was improved than the Hollow and 2D group, and was closer to those in the ANG group (Fig. 6a).

At the 4th week after transplantation, there was no statistical difference in SFI between the four groups. The SFI of 2D, Microtissue, and ANG group were significantly higher than Hollow group at the 8th week after transplantation, and there was no statistical difference between Microtissue group and 2D group. At the 12th week after transplantation, the SFI of the four groups was further improved. The Microtissue group (− 50.86 ± 4.42) was significantly better (*p* < 0.0001) than 2D group (− 65.42 ± 2.36), and there was no statistical difference between the Microtissue group and ANG group (− 47.43 ± 2.85) (Fig. 6c).

### Electrophysiology

At the 12th week after transplantation, representative CMAPs oscillograms of four groups are shown in Fig. 6b. The peak amplitude ratio of CMAPs in the Microtissue group (52.75 ± 1.21%) was significantly higher (*p* < 0.01) than the 2D group (42.82 ± 3.45%), and there was no statistical difference between Microtissue group and ANG group (57.00 ± 7.05%), 2D group and Hollow group (38.79 ± 3.31%), respectively (Fig. 6d). The latency ratio of CMAPs in the Hollow group (2.95 ± 0.17) was significantly longer (*p* < 0.0001) than the 2D group (2.02 ± 0.11), Microtissue group (2.02 ± 0.12), and ANG group (1.75 ± 0.28), and there was no statistical difference between the latter three groups (Fig. 6e).

### Histological evaluation of nerve grafts

**Fig. 7:**
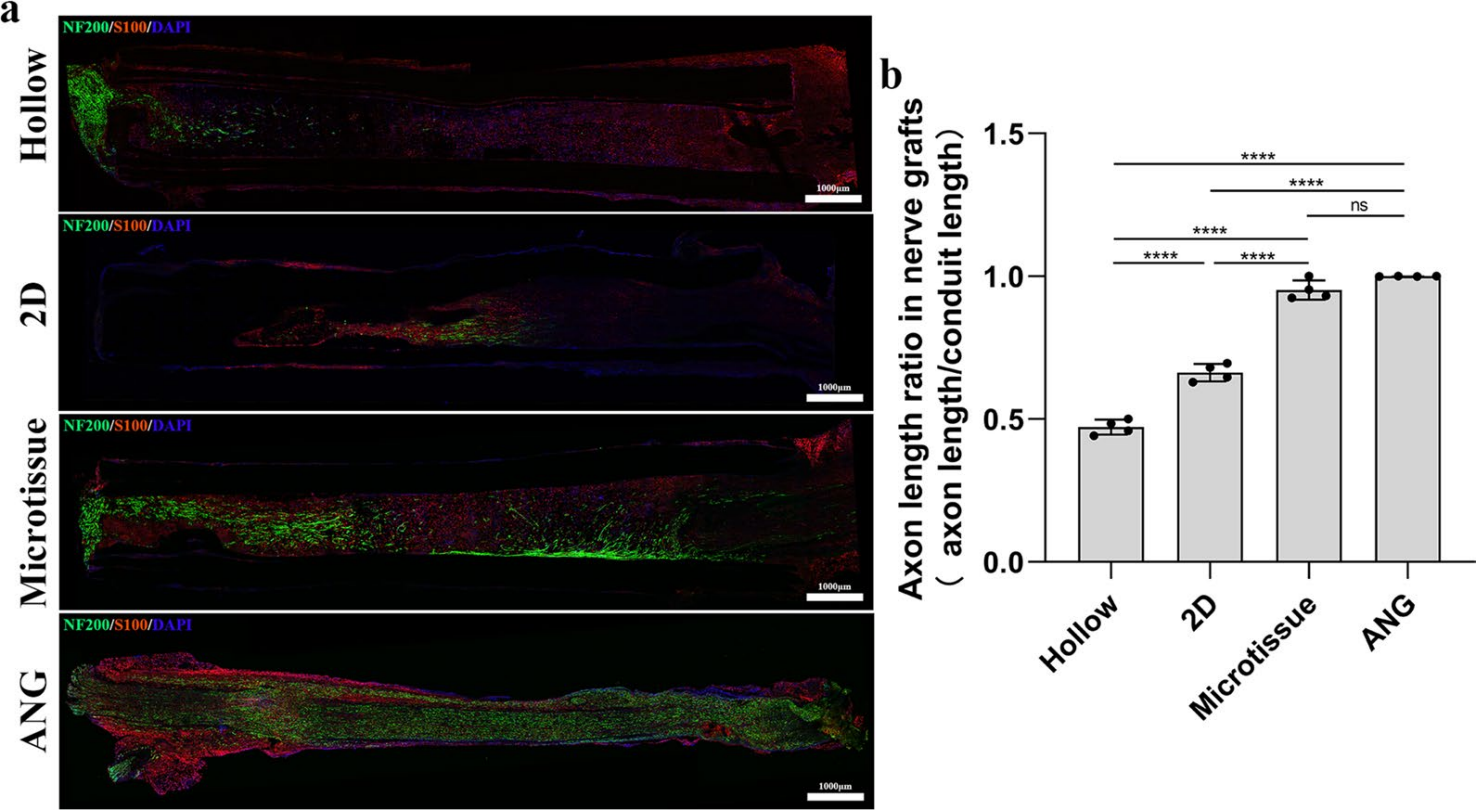
Histological evaluation of nerve grafts at the 4th week after transplantation. **a** The axons exhibited green fluorescence, the myelin sheath exhibited red fluorescence, and all nuclei showed blue fluorescence. Scale bars: 1000 μm. **b** Axon length ratio (axon length/conduit length) in the Microtissue group was significantly higher (*p* < 0.0001) than the 2D group and Hollow group, and there was no statistical difference between Microtissue group and ANG group

To observe the nerve growth in the early stage, the nerve grafts of four groups were sliced longitudinally into 9-μm slices at the 4th week after transplantation, and then immunofluorescence staining was performed on the samples. Immunofluorescence analysis showed that axons exhibited green fluorescence, the myelin sheath exhibited red fluorescence, and all nuclei showed blue fluorescence (Fig. 7a). Axon length ratio (axon length/conduit length) in the Microtissue group (95.23 ± 3.39%) was significantly higher (*p* < 0.0001) than the 2D group (66.28 ± 3.05%) and Hollow group (47.17 ± 2.63%), and there was no statistical difference between Microtissue group and ANG group (100%) (Fig. 7b).

**Fig. 8:**
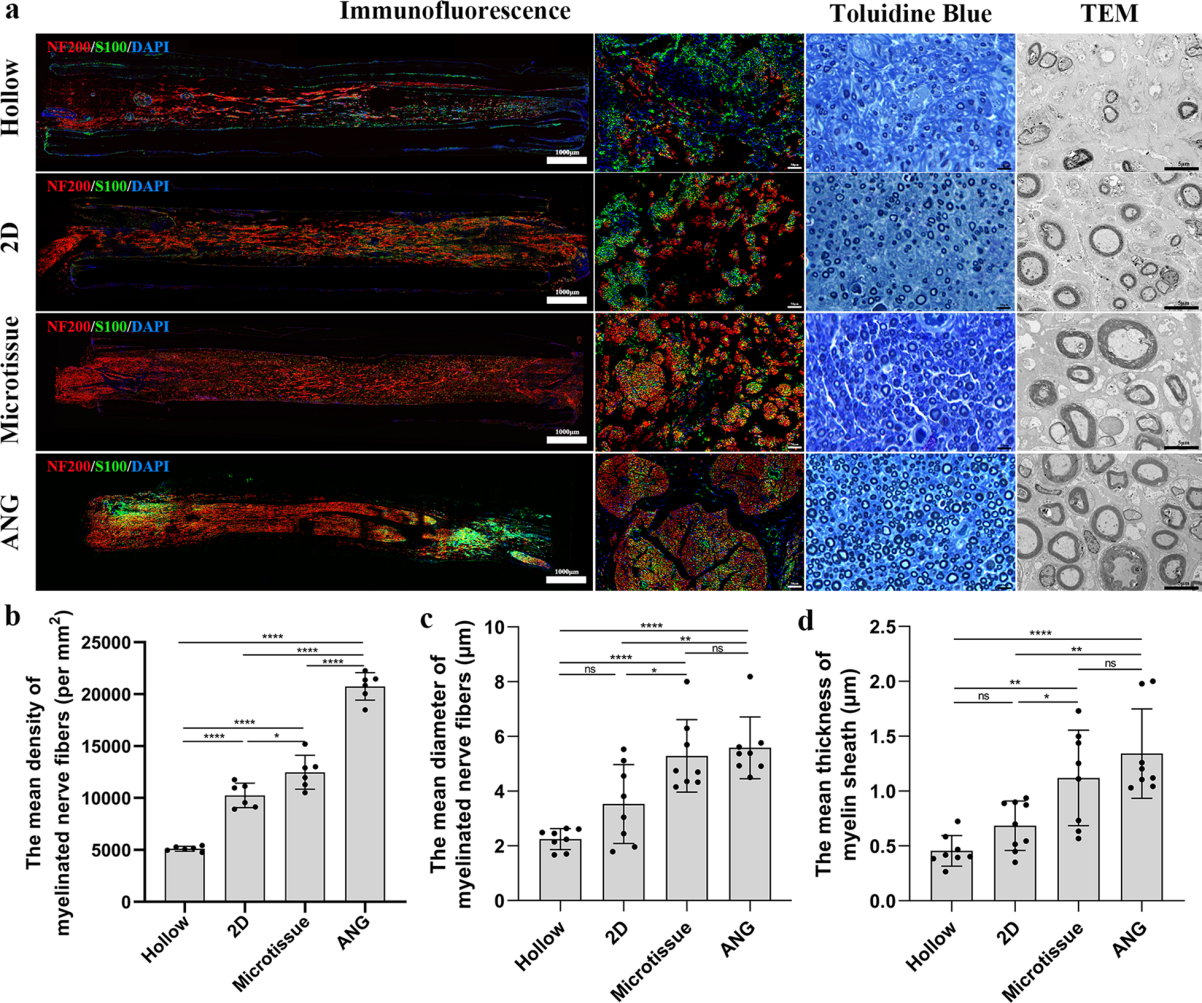
Histological evaluation of nerve grafts at the 12th week after transplantation. **a** In the immunofluorescence photographs (the first column, longitudinal sections of the nerve grafts; the second column, transversal sections of the nerve grafts middle segment), the axons exhibited red fluorescence, the myelin sheath exhibited green fluorescence, and all nuclei showed blue fluorescence. The photographs in the third and fourth columns show Toluidine Blue staining and transmission electron microscopy (TEM) images, respectively, of nerve grafts distal segment. Scale bars: 1000 μm (leftmost column), 50 μm (second column), 10 μm (third column) and 5 μm (rightmost column). **b** The mean density of myelinated nerve fibers in Microtissue group was significantly higher than the 2D group (*p* < 0.05). **c, d** The mean diameter of myelinated nerve fibers and mean thickness of myelin sheaths in the Microtissue group were significantly higher (*p* < 0.05) than those in the 2D group, and there was no statistical difference between Microtissue group and ANG group

At the 12th week after transplantation, immunofluorescence analysis showed that axons exhibited red fluorescence, the myelin sheath exhibited green fluorescence, and all nuclei showed blue fluorescence. Axonal growth was observed throughout the nerve graft in all four groups; however, the density of axons in nerve grafts of the Microtissue group was higher than in 2D group and the Hollow group and was closer to those in the ANG group (Fig. 8a). Toluidine Blue staining and TEM results of regenerative nerves located at the middle section of never graft showed that the myelinated nerve fibers and myelin sheaths in the Microtissue group and ANG group were thicker than Hollow group and 2D group (Fig. 8a).

The mean density of myelinated nerve fibers in Microtissue group (12,477 ± 1642/mm^2^) was significantly higher than the 2D group (10,248 ± 1182/mm^2^) and Hollow group (5111 ± 239/mm^2^) (microtissue VS 2D, *p* < 0.05; microtissue VS Hollow, *p* < 0.0001), at the 12th week after transplantation. Similarly, the density of myelinated nerve fibers in the ANG group (20,749 ± 1322/mm^2^) was much higher (*p* < 0.0001) than in the Microtissue group (Fig. 8b).

The mean diameter of myelinated nerve fibers and mean thickness of myelin sheaths in the Microtissue group (5.29 ± 1.33 μm in diameter and 1.12 ± 0.44 μm in thickness) were significantly higher (*p* < 0.05) than those in the Hollow group (2.24 ± 0.38 μm in diameter and 0.46 ± 0.14 μm in thickness) and 2D group (3.53 ± 1.44 μm in diameter and 0.68 ± 0.22 μm in thickness), and there was no statistical difference between Microtissue group and ANG group (5.59 ± 1.13 μm in diameter and 1.34 ± 0.41 μm in thickness) (Fig. 8c, d).

### Histological evaluation of the gastrocnemius

**Fig. 9:**
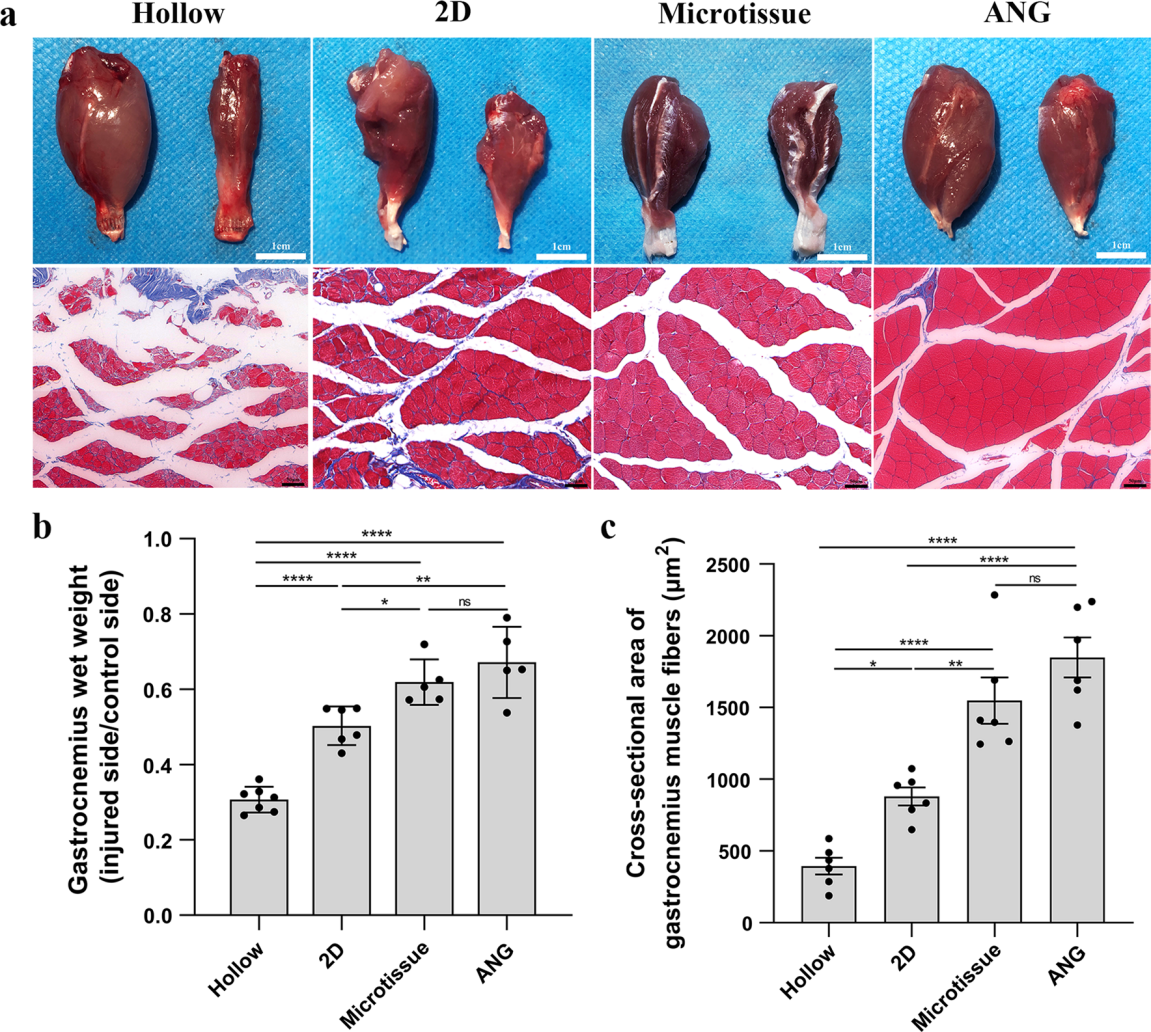
Histological evaluation of the gastrocnemius. **a** Representative results of the gastrocnemius on the injured side shows visible atrophy in the Hollow group and 2D group compared with the normal side at the 12th week after transplantation. In contrast, the other two groups were less pronounced (up row). Masson staining of the gastrocnemius muscle in the four groups showed the hyperplasia of blue-stained collagenous between the red-stained muscle fibers (bottom row). Scale bars: 1 cm (up row) and 50 μm (bottom row). **b** The gastrocnemius wet weight recovery ratio in the Microtissue group was significantly higher than the 2D group (*p* < 0.05), and there was no statistical difference between the Microtissue group and ANG group. **c** The mean cross-sectional area of gastrocnemius fibers in the Microtissue group was significantly greater than the 2D group (*p* < 0.01), and there was no statistical difference between the Microtissue group and ANG group

At the 12th week after transplantation, the degree of gastrocnemius atrophy in the Hollow group and 2D group was significantly higher than the Microtissue group and ANG group according to the general view of gastrocnemius (Fig. 9a). The gastrocnemius wet weight recovery ratio in the Microtissue group (61.94 ± 6.03%) was significantly higher than the 2D group (50.33 ± 5.12%) and Hollow group (30.73 ± 3.40%) (microtissue VS 2D, *p* < 0.05; microtissue VS Hollow, *p* < 0.0001), and there was no statistical difference between the Microtissue group and ANG group (67.18 ± 9.48%) (Fig. 9b). Masson staining of the gastrocnemius muscle in the four groups showed the hyperplasia of blue-stained collagenous between the red-stained muscle fibers (Fig. 9b). The mean cross-sectional area of gastrocnemius fibers in the Microtissue group (1548.00 ± 394.00μm^2^) was significantly greater than the 2D group (880.90 ± 153.00μm^2^) and Hollow group (394.20 ± 143.10μm^2^) (microtissue VS 2D, *p* < 0.01; microtissue VS Hollow, *p* < 0.0001), and there was no statistical difference between the Microtissue group and ANG group (1849.00 ± 343.20μm^2^) (Fig. 9c).

## Discussion

In recent years, tissue-engineered microtissue strategy has made significant research progress in many research fields. In bone tissue engineering, Totaro et al. [49] mixed human mesenchymal stem cells (hMSCs) and polycaprolactone (PCL)—hydroxyapatite (HA) microscaffolds and placed them in a rotating flask for dynamic culture to construct bone microtissue. Compared with 2D culture, hMSCs in the microtissue have robust osteogenic differentiation ability. In cartilage tissue engineering, Wang et al. [50] cultured nanofiber microcarrier mimicking ECM and bone marrow-derived mesenchymal stem cells (BMSCs) into functional cartilage microtissues under microgravity condition, and implanted the microtissues directly into the knee cartilage defects of Sprague–Dawley rats, achieving promising repair effects. In myocardial tissue engineering, the researchers encapsulated induced pluripotent stem cells (iPSCs) into the omental ECM hydrogel in microfluidic system to make myocardial microtissues. The injection of microtissue into the gastrocnemius muscle did not damage the cellular activity within the microtissue [51]. Similarly, the application of microtissue strategy in adipose tissue engineering [22, 28], vascular tissue engineering [41, 42], oncology research [43, 44] and other fields has also achieved extensive achievements. However, the application of microtissue strategy in neural tissue engineering is rare, we used microtissue in the current study to conduct research on nerve injury repair.

At present, there are four main approaches of microtissue construction, including hanging-drop cell culture [52], spheroid-based cell culture [22], microcarrier or microscaffold-based cell culture [19, 24] and microgel or microsphere-based cell culture [53]. The first two kinds of microtissue are formed by cell self-aggregation with microgravity or low adherent cell culture materials, and the latter two kinds of microtissue are formed using microcarriers or microgel loaded cells. In our study, to explore the direct interaction between microtissue and SCs or DRG in vitro, we chose the hanging-drop method and spheroid method to construct microtissue. However, we found that the microtissue constructed by the hanging-drop method was loose, and easy to disintegrate with the culture time, so we chose spheroid method to construct the microtissue for subsequent experiments.

An important component of tissue-engineered microtissue is the seed cell. MSCs, derived from pluripotent adult precursors with self-renewal ability in the germ layer of the mesenchymal layer, can promote nerve regeneration by inducing the secretion of various neurotrophic factors and are promising seed cells for tissue engineering [45, 54]. Although BMSCs have been used to repair tissue damage, including that of the peripheral nerve, the low yield and invasiveness in the extraction process seriously limit the clinical application of BMSCs [55]. Compared with BMSCs, ASCs with lower invasiveness in the extraction process, higher yield and faster proliferation rate in vitro are a better choice for tissue engineering [56]. Moreover, the application of ASCs has achieved a promising repair effect in PNI [57–60]. Taken together, these advantages indicated that ASCs are a good choice for the construction of microtissues.

Regarding the preparation of microtissues, the number of cells contained in each microtissue varies greatly, from 1440 to 40,000 cells per microtissue [22, 61, 62]. Paradoxically, the function of microtissue is poor when there are too few cells, and cell necrosis will appear in the depths of microtissue when there are too many cells. To explore the appropriate number of cells in microtissue, we constructed microtissues using 5000, 7500, and 10,000 cells, respectively. After three days of culture, live/dead staining was performed on the microtissues, showing that most of the cells in the three types of microtissues remained viable. To maximize the function of the microtissues, we selected microtissues containing 10,000 cells for subsequent experiments. With the culture time, the diameter of microtissues decreased gradually and presented a compacted state, which was consistent with the research results of Colle et al. [28].

ECM is a 3D network without cells. Its main components are collagen, proteoglycan/glycosaminoglycan, elastin, fibronectin, laminin and other glycoproteins [63]. Fibronectin can be expressed in the ECMs of various important functional cells in vertebrates [64]. Fibronectin molecules secreted by cells are assembled into supramolecular fibers, connected to form a fibrous network, and cells adhere to the fibrous network [65]. Laminin is a large heterotrimeric glycoprotein. Laminin molecules participate in the composition of ECM and cell adhesion through the interaction with other ECM components and resident cells [66, 67]. In the current study, immunofluorescence analysis showed that red-stained fibronectin and green-stained laminin were all over the microtissue, indicating that there was ECM in the microtissue, and it was because of the existence of ECM that the cells could aggregate into microtissue.

qRT-PCR and ELISA results indicated that BDNF expression at transcriptional and translational levels in the Microtissue group was significantly higher than the 2D group. BDNF can enhance the intrinsic capacity of axonal regeneration. On the one hand, BDNF can activate the BDNF/TrkB pathway to form the actin waves; on the other hand, BDNF can improve cAMP levels and upregulates the CREB-cjun-STAT3-Gap-43 pathway to improve the ability of axon growth [68, 69]. The neural electrophysiological evaluation showed that the peak amplitude of CAMPs in the Microtissue group with a higher expression level of BDNF was significantly higher than the 2D group and the Hollow group and similar to that in the ANG group (Fig. 7c). In addition to promoting axonal growth, BDNF also plays an important role in the proliferation of Schwann cells. BDNF can regulate the proliferation of SCs to promote remyelination [70]. A previous study indicated that long-chain non-coding RNA MALAT1 was enhanced after peripheral nerve injury, which increased the expression and secretion of BDNF through sponging miR-129-5p, thus promoting proliferation and migration of Schwann cells [71]. Under the effect of BDNF, the proliferation rate of SCs in the Microtissue group was significantly higher than the 2D group in the transwell system.

Similarly, the expression of VEGF in both transcriptional and translational levels in the Microtissue group was higher than in the 2D group. Although VEGF can promote vasculogenesis and angiogenesis, increasing evidence suggests that VEGF plays a key role in the nervous system, such as promoting axon growth in various neurons [72]. VEGF-A-165 is the dominant isoform in most mammalian tissues, two isoform families are produced by alternative splicing: VEGF-A-165a and VEGF-A-165b, and VEGF-A-165a have nerve regeneration potential [73, 74]. Previous studies have shown that VEGF has a significant effect on the axonal growth of DRG [75]. Moreover, VEGF attracts and influences the speed and size of the growth cone during nerve regeneration [76]. Ruiz de Almodovar et al. showed that VEGF expressed and secreted by floor plate and VEGF receptor Flk1 expressed and secreted by commissural neurons jointly guided axonal growth in a spinal cord ventral midline model system [77]. Taken together, VEGF can promote axon growth and guide axon growth direction with its receptor Flk1. In the direct co-culture system of microtissue and DRG, DRG axons are much longer and grow toward microtissue, which may be due to the large amount of VEGF secreted by microtissue (Fig. 5).

qRT-PCR results indicated that anti-inflammatory cytokines (IL-4, IL-10, and IL-13) expression at the transcriptional level in the Microtissue group was significantly higher than the 2D group. We performed ELISA for IL-13, and the results showed that the secretion level of IL-13 in the Microtissue group was significantly higher than the 2D group. In the early stage of injury, the inflammatory reaction can better limit the necrotic and apoptotic cells or foreign bodies in a certain area to prevent the expansion of lesions. However, in the later stage, inflammatory cells often overreact, infiltrate a large number of local areas, release a large number of pro-inflammatory factors, but further aggravate tissue damage [78]. Compared with 2D ASCs, microtissues can secrete various anti-inflammatory factors and better inhibit the inflammatory response, which may reduce tissue damage and achieve the purpose of repair. In addition, IL-4 has been reported to regulate cell survival, proliferation, and branching in the nervous system, and promote peripheral axonal regeneration [79].

ELISA results indicated that NGF expression at translational level in the Microtissue group was significantly higher than those in the 2D group. NGF is widely distributed in various tissues and organs of the body, the first isolated neurotrophic factor that promotes axon growth and elongation [80]. A previous study indicated that NGF could promote axon growth by abolishing neuronal growth cone-collapsing factor semaphorin3A (Sema3A)-induced axon growth inhibition [81]. Moreover, exogenous NGF can activate the autophagy of dedifferentiated SCs, accelerate the clearance and phagocytosis of myelin fragments, promoting axon and myelin regeneration [80]. In addition to promoting axon growth, NGF may also be involved in myelin formation. Previous studies reported that NGF may induce remyelination through its activity on congenital oligodendrocyte precursors, and can act directly on various cells involved in myelination (such as Schwann cells) [82, 83]. This explains why the density of myelinated nerve fibers and the thickness of myelin sheath in the Microtissue group were significantly higher than those in the Hollow and 2D groups. In addition, better myelin regeneration leads to a shorter latency of CAMPs in the Microtissue group.

qRT-PCR and ELISA results indicated that 3D-cultured ASCs can express and secrete more nutritional factors and anti-inflammatory factors compared with 2D culture, but the underlying mechanism remains unclear. There are two possible causes of altered expression of nutritional and anti-inflammatory factors: First, 3D culture changes the expression of adhesion molecules in MSCs. The surrounding microenvironment is known to influence the fate of mesenchymal stem cells, which are sensed by adhesion complexes on cell membranes and conducted through actin cytoskeleton tension [84]. 2D-cultured MSCs had abundant integrin β1 and Vinculin distribution, which resulted in high contraction of the attached cytoskeleton. However, integrin β1 and Vinculin levels were significantly decreased in 3D-cultured MSCs, and integrin-based cell–matrix adhesion was decreased, while cadherin-based cell–cell interaction was increased [85]. Second, 3D culture alters the cytoskeleton of MSCs. Cytoskeleton maintains the cell morphology according to the mechanical and biochemical characteristics of microenvironment [86]. The spheroids were digested by trypsin and then the MSCs were reattached to the culture plate, resulting in uniformed spindle-like cells with small size, less spreading with sharp edges [87, 88]. Zhou et al. [85] have shown that the cytoskeleton of 2D MSCs formed an extended network structure and arranged in a flat shape, while in 3D culture, the cytoskeleton was loose in tension. Moreover, the rearrangement of actin cytoskeleton is largely responsible for the continuous effect of 3D culture on cell size and morphology. In conclusion, the increased expression levels of nutritional factors and anti-inflammatory factors in 3D-cultured cells may be related to the expression of cell adhesion molecules and the changes of cytoskeleton. The specific mechanism is still unclear and needs further exploration.

In vitro, we used the transwell system to indirectly co-culture DRG with microtissues or monolayer cells. The upper insert and lower chamber were separated by a 0.4-μm polyester membrane, allowing the upper and lower chambers to exchange cytokines and block cell contact. Therefore, we used the transwell system to study the difference between the two groups of paracrine effects on promoting DRG axon growth. Compared with the traditional single-layer cultured cells, the microtissues can secrete more BDNF, NGF, VEGF and IL-13, all of them can promote the growth of axons, so the length of axons in the Microtissue group is significantly longer than the 2D group. The axonal growth-promoting effects of above neurotrophic factors, angiogenic factors and anti-inflammatory factors have been verified in vivo. In the early stage of transplantation (4 weeks), axons in the Microtissue group penetrated the whole nerve graft, whereas the ratio of axon length in the 2D group and the Hollow group was only 66% and 47%. In the later stage of transplantation (12 weeks), although the axons of all four groups penetrated the nerve grafts, immunofluorescence analysis showed that the axon density of the Microtissue group was significantly higher than the 2D group and the Hollow group. In addition, higher SFI and better gastrocnemius recovery also indicated that the Microtissue group had achieved better nerve repair effect than the 2D group and Hollow group.

Microtissue can secrete many neurotrophic factors, angiogenesis factors and anti-inflammatory factors to promote nerve regeneration. In addition to the paracrine effect, whether microtissue can directly act on DRG or Schwann cells is also worthy to explore. To solve this problem, we conducted a direct contact co-culture of microtissues with DRG or Schwann cells to explore cell-to-cell interaction between them. After 7 days of direct co-culture between microtissues and DRG, the axon length of DRG was much longer than the indirect co-culture system, and the axon grew in the direction of microtissues. As discussed earlier, VEGF secreted by microtissues can promote and guide the growth of axons. In the indirect co-culture system, VEGF was evenly dispersed in the medium, while in the direct co-culture system, the instantaneous concentration of VEGF around microtissues was higher than that in other areas, so axons grew in the microtissues direction. After 3 days of direct co-culture between microtissues and SCs, we found an interesting phenomenon. Red and green double-stained cells appeared around the red-stained microtissues and green-stained Schwann cells. These cells were spindle-like in morphology, which is similar to SCs, and the two kinds of cells appeared to have undergone cytoplasmic exchange. This phenomenon may have implications for the research of the outcome of stem cells implantation in vivo, and the causes and mechanisms of direct cellular interactions need to be explored further.

## Conclusion

The present study results indicated that compared with the same number of 2D-cultured cells, microtissue could secrete more nerve regeneration related cytokines to promote Schwann cell proliferation and axon growth. Moreover, we found an interesting phenomenon in the direct co-culture system of microtissue and DRG or SCs. Axons of DRG grown in the direction of microtissue, and there seemed to be the cytoplasmic exchange between SCs and ASCs around microtissue. Furthermore, microtissues could repair sciatic nerve defects in rat models more effectively than traditional 2D-cultured ASCs. Thus, tissue-engineered microtissue is a promising strategy for stem cell culture and therapy in nerve tissue engineering.

## Supplementary Information

**Additional file 1: Figure S1.** The Transwell® system. DRGs or SCs were seeded in the lower chambers of the system, which consists of six inserts, each containing a polyester membrane with a diameter of 24 mm and an aperture of 0.4 μm. The microtissues or ASCs were seeded in the upper inserts of the Transwell® system.

**Additional file 2: Figure S2.** Characterization of ASCs. The positive rates of mesenchymal stem cell surface marker antigens CD73, CD90 and CD105 and hematopoietic stem cell surface marker antigens CD34 and CD45 in P3 generation ASCs.

**Additional file 3: Figure S3.** The diameter variation of microtissue containing varying numbers of cells. The microtissue diameter decreased with the extension of culture time. However, there was no statistical difference in the diameter of microtissues with different cell numbers (5000 cells/microtissue or 7500 cells/microtissue or 10,000 cells/microtissue).

## Data Availability

All data generated or analyzed during this study are included in this published article.

## Abbreviations

PNI: Peripheral nerve injury
ASCs: Adipose-derived mesenchymal stem cells
BMSCs: Bone marrow-derived mesenchymal stem cells
2D: Two-dimension
3D: Three-dimension
ECM: Extracellular matrix
MSCs: Mesenchymal stem cells
DRG: Dorsal root ganglion
SCs: Schwann cells
SD: Sprague–Dawley
DMEM/F-12: Dulbecco’s modified Eagle’s medium/Ham’s F-12
α-MEM: α-Modified minimal essential medium
FBS: Fetal bovine serum
GFP: Green fluorescent protein
DAPI: 4′,6-Diamidino-2-phenylindole dihydrochloride
qRT-PCR: Quantitative reverse transcription polymerase chain reaction
GAPDH: Glyceraldehyde 3-phosphate dehydrogenase
BDNF: Brain-derived neurotrophic factor
VEGF: Vascular endothelial growth factor
IL-4: Interleukin-4
IL-10: Interleukin-10
IL-13: Interleukin-13
ELISA: Enzyme-linked immunosorbent assay
NGF: Nerve growth factor
cDNA: Complementary DNA
PBS: Phosphate-buffered saline
NF200: Neurofilament protein 200
PCL: Polycaprolactone
SFI: Sciatic nerve function index
CMAPs: Compound muscle action potentials
TEM: Transmission electron microscopy
RH: Right hind
LH: Left hand
ANG: Autologous nerve graft
HA: Hydroxyapatite
hMSCs: Human mesenchymal stem cells
iPSCs: Induced pluripotent stem cells
Sema3A: Semaphorin3A
