# Supplementary figures and images for: Functional tissue-engineered microtissue formed by self-aggregation of cells for peripheral nerve regeneration
Source: Stem Cell Res Ther. 2022 Jan 10;13:3. doi: 10.1186/s13287-021-02676-0 (PMC8744299; doi:10.1186/s13287-021-02676-0)

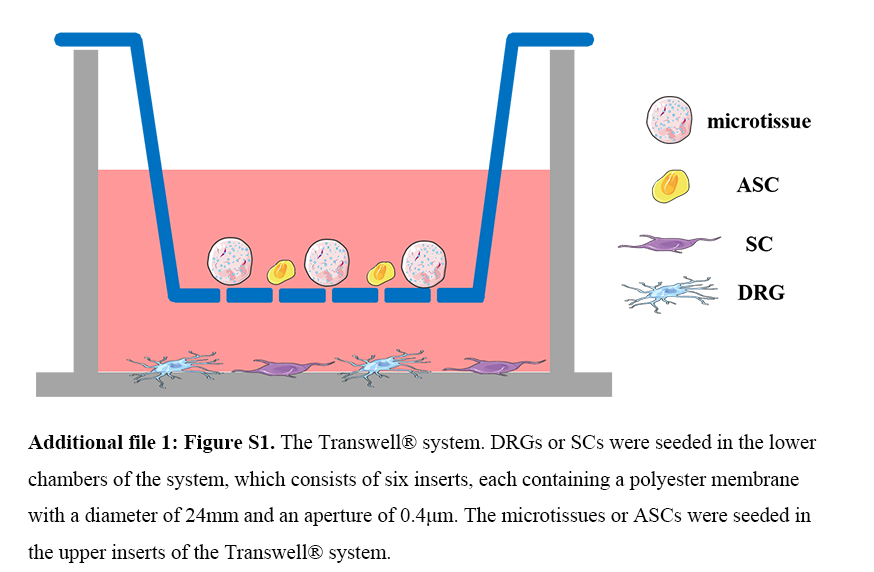

Supplement: Supplementary file 1 — Additional file 1: Figure S1. The Transwell® system. DRGs or SCs were seeded in the lower chambers of the system, which consists of six inserts, each containing a polyester membrane with a diameter of 24 mm and an aperture of 0.4 μm. The microtissues or ASCs were seeded in the upper inserts of the Transwell® system. [file 13287_2021_2676_MOESM1_ESM.tif]

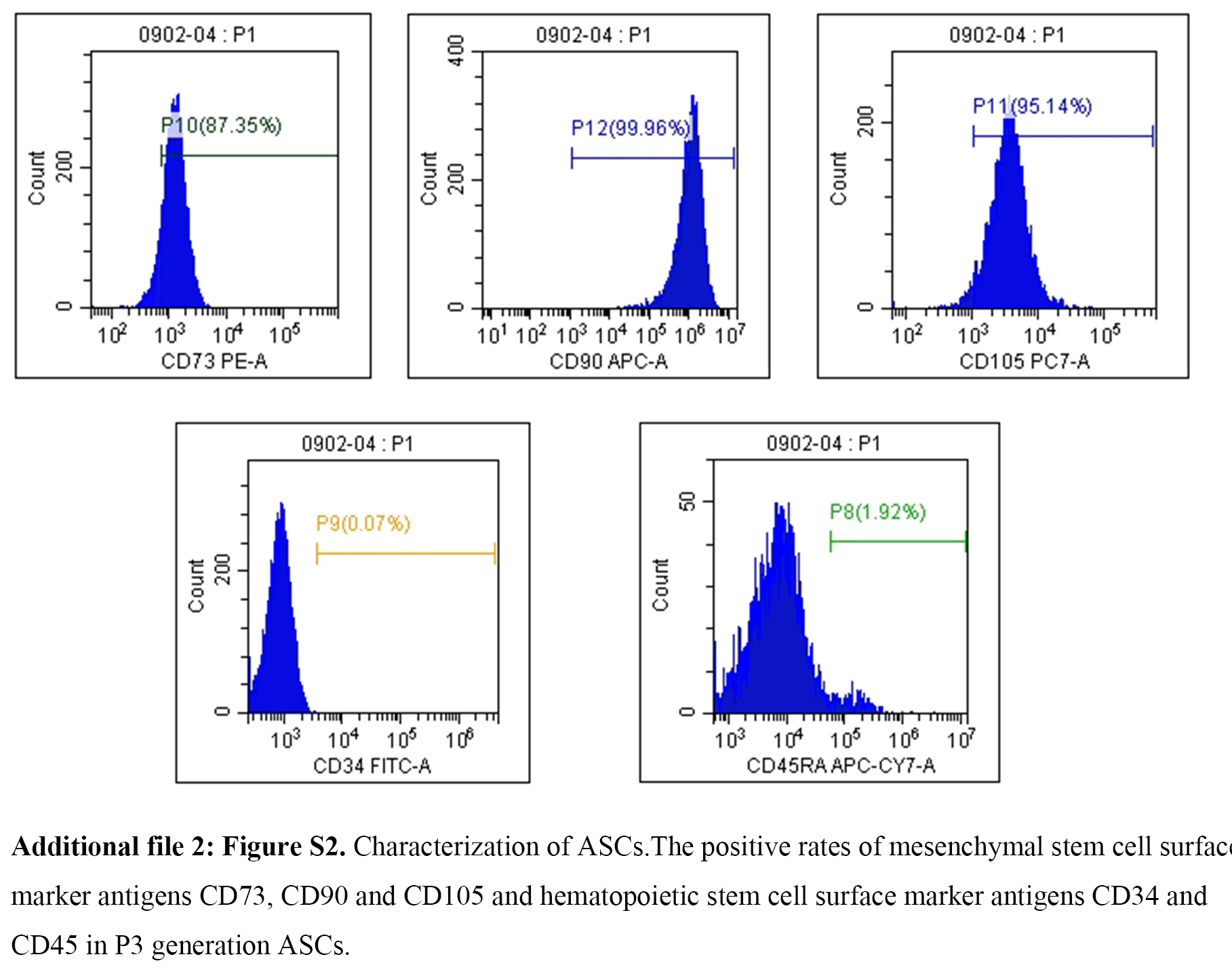

Supplement: Supplementary file 2 — Additional file 2: Figure S2. Characterization of ASCs. The positive rates of mesenchymal stem cell surface marker antigens CD73, CD90 and CD105 and hematopoietic stem cell surface marker antigens CD34 and CD45 in P3 generation ASCs. [file 13287_2021_2676_MOESM2_ESM.tif]

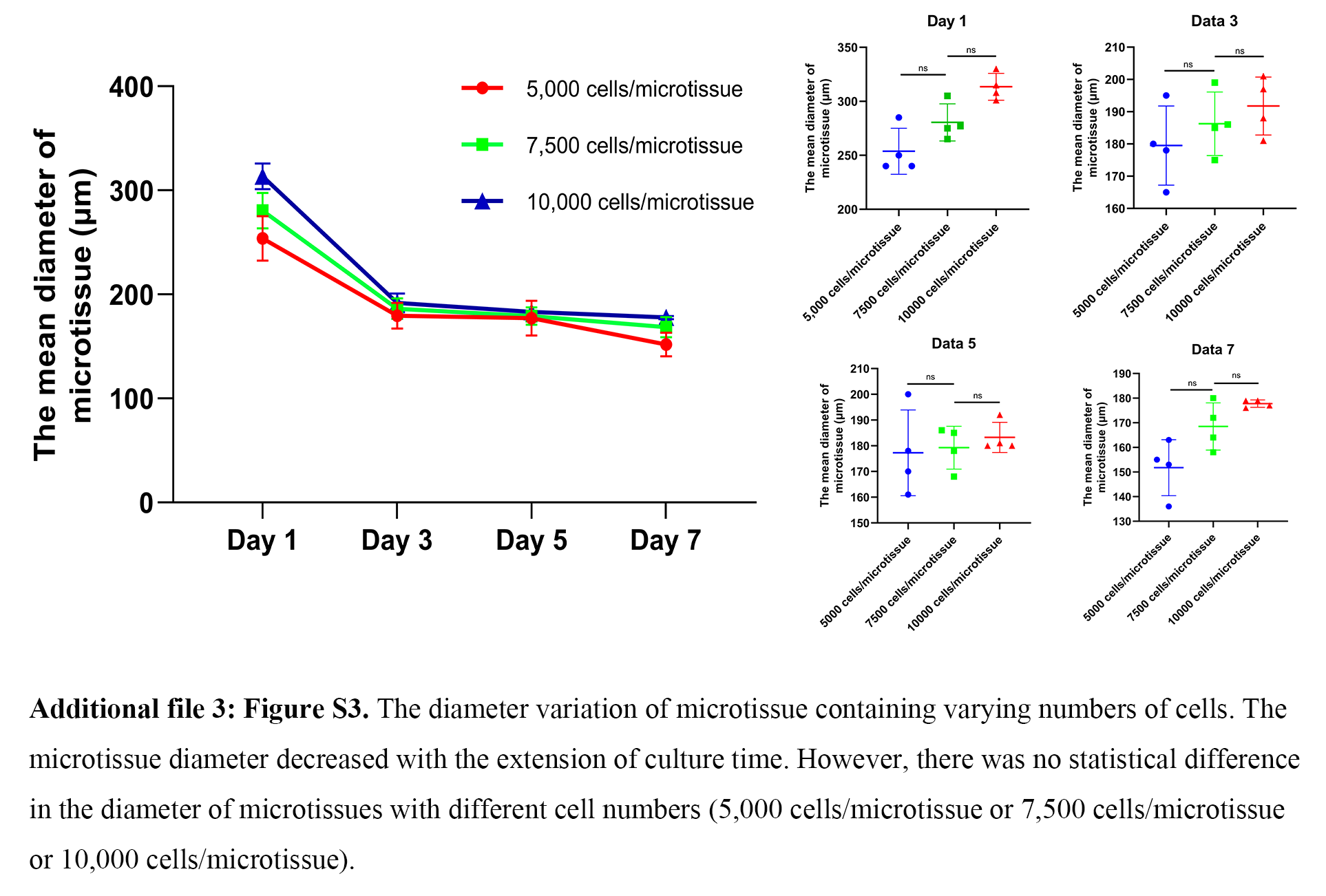

Supplement: Supplementary file 3 — Additional file 3: Figure S3. The diameter variation of microtissue containing varying numbers of cells. The microtissue diameter decreased with the extension of culture time. However, there was no statistical difference in the diameter of microtissues with different cell numbers (5000 cells/microtissue or 7500 cells/microtissue or 10,000 cells/microtissue). [file 13287_2021_2676_MOESM3_ESM.tif]
